# A case of rare lumbar intradural tumor: paraganglioma

**DOI:** 10.1093/jscr/rjae054

**Published:** 2024-02-13

**Authors:** Ismail Ertan Sevin, Onur Davut Dağ, Aslı Kahraman, Hasan Kamil Sucu

**Affiliations:** Department of Neurosurgery, Izmir Katip Celebi University, Ataturk Training and Research Hospital, 35360 Izmir, Turkey; Department of Neurosurgery, Izmir Katip Celebi University, Ataturk Training and Research Hospital, 35360 Izmir, Turkey; Department of Pathology, Izmir Katip Celebi University, Ataturk Training and Research Hospital, 35360 Izmir, Turkey; Department of Neurosurgery, Izmir Katip Celebi University, Ataturk Training and Research Hospital, 35360 Izmir, Turkey

**Keywords:** case report, lumbar, paraganglioma, surgery

## Abstract

Lumbar paragangliomas are rare neuroendocrine neoplasms arising from specialized neural crest cells in the cauda equina/filum terminale region. They are difficult to diagnose radiologically and can be difficult to treat surgically if they secrete catecholamines. A 38-year-old woman presented with three and a half years of increasing lower back and sacrum discomfort. Her neurological examination was normal. The MRI revealed an L4 intradural lesion that was compressing the cauda equina. A total tumor resection was conducted. The paraganglioma was diagnosed by the pathology report. Paragangliomas should be considered in the differential diagnosis of intradural masses of the lumbar spine.

## Introduction

Due to the widespread distribution of paraganglionic tissue, paragangliomas may arise in any part of the body. Still, tumors of the carotid body and glomus jugulare account for more than 90% of cases [1]. Spinal paragangliomas are uncommon [1] and originate from specialized neural crest cells in the cauda equina/filum terminale region, where they are classified as ‘Cauda equina neuroendocrine tumour’ in the World Health Organization (WHO) 2021 Central Nervous SystemTumours classification. These tumors are considered grade 1. There are slightly more than 200 cauda equina paragangliomas reported in the literature. Due primarily to magnetic resonance imaging (MRI), reporting frequency has increased in recent years [2]. We would like to discuss cauda equina paragangliomas with the presentation of a case in which we performed surgery and draw attention to the fact that they should be considered in the differential diagnosis.

## Case report

**Figure 1:**
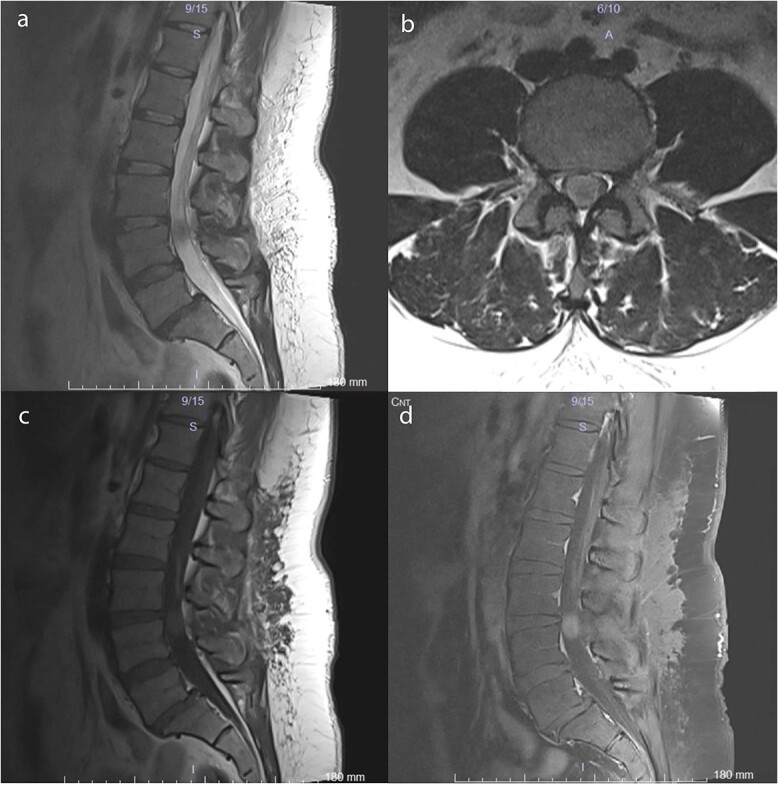
Preoperative MRI scans demonstrate a 17 × 12 × 15 mm intradural lesion posterior to the L3–4 disc and upper part of the L4 corpus. (a) The edge of the hypointense lesion is not so sharp in the midsagittal *T*_2_-weighted image. (b) In contrast to the sagittal *T*_2_-weighted image, the tumor appears to be hyperintense in the axial *T*_2_-weighted image. Because the nerve fibers are compressed by the tumor toward the dura CSF is not visible. (c) Midsagittal *T*_1_-weighted image shows a barely noticeable intradural tumor that is isointense with the vertebral body. (d) On midsagittal contrast-enhanced *T*_1_-weighted image, the tumor was homogeneously enhanced and became prominent.

A 38-year-old female with a 1-month history of tailbone pain was admitted to the hospital. No significant medical history was present. On examination, all lower extremities were rated 5/5 for motor function. The senses and reflexes were symmetrical and intact. The patient did not report any problems with urinary or fecal continence. MRI revealed an intradural lesion at the L4 level (Fig. 1).

**Figure 2:**
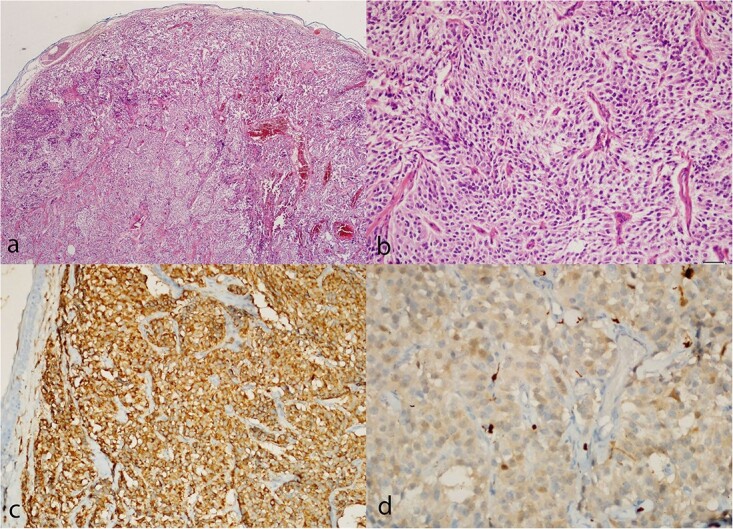
Paraganglioma histology. (a, b) Hematoxylin and eosin sections show a well-circumscribed tumor composed of uniform chief cells arranged in a typical acinar pattern (Zellballen). (c) Immunohistochemistry with the antibodies to chromogranin A highlights the chief cells. (d) Small sustentacular cells incompletely surround some acini, as highlighted with the S-100 antibody.

The patient underwent surgery 20 days after admission. L4 total laminectomy was performed, and the dura was opened. The encapsulated, soft, and gray-colored tumor originating from the filum terminale was excised in its entirety by severing the filum terminale at the tumor’s cranial and caudal margins. The dura was closed primarily. The surgery was uneventful, and there was no hypertensive crisis. The paraganglioma was diagnosed by the pathology report. Histopathological examination of hematoxylin and eosin sections revealed a well-circumscribed tumor constituted of uniform, acinar-like chief cells (Zellballen pattern) (Fig. 2a and b). Positive staining for the neuroendocrine markers synaptophysin and chromogranin A was observed in tumor cells (Fig. 2c). Additionally, S100 positivity was observed in the encircling sustentacular cells of the tumor (Fig. 2d).

**Figure 3:**
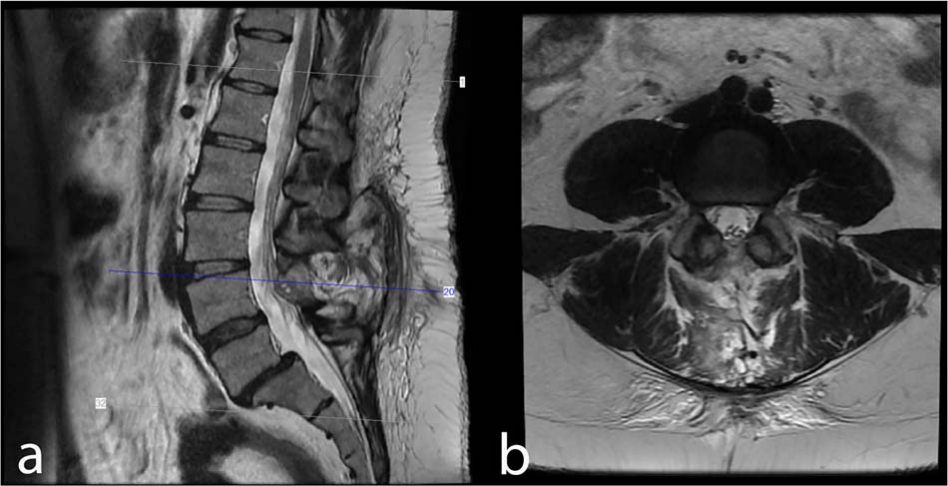
Forty-three month postoperative (a) midsagittal and (b) axial MRI images show no residual/recurrent intradural lesion.

The patient exhibited no neurological deficits after surgery and experienced relief from tailbone pain. In the patient’s 43-month follow-up, she exhibited no symptoms or indications. The control MRI revealed no remnants or recurrence (Fig. 3).

## Discussion

The paraganglia is a widely distributed neuroendocrine tissue that originates from the neural crest. They can be classified as either sympathetic or parasympathetic paraganglia. From either form of paraganglia, paragangliomas can develop [3].

The peak incidence of lumbar spinal paraganglioma occurs during the fifth decade, with the average age of presentation being 47 years. With a male-to-female ratio of 1.54:1, there is a male predominance [4]. Thus, our 38-year-old female patient did not exhibit the typical epidemiological features of paraganglioma.

Paragangliomas are predominantly benign and have a slow rate of growth. They are usually discovered by accident or due to mass effect [5]. It was reported that the most common clinical symptom was low back pain with a rate of 79.8%, as in our patient if we consider tailbone pain as low back pain [4]. However, sciatica, which was reported in 53.2% of patients [4], was absent in our patients. Motor and sensory deficits, which were previously reported to be uncommon (24.9% and 22%, respectively) [4], were also absent in our patient. In addition, other rarely reported findings, such as urinary or fecal incontinence, impotence, and pseudo claudication (11.4, 1.0, and 0.7%, respectively) [4], were not present in our patient. Only 2.4% of reported lumbar paragangliomas exhibited catecholamine-secreting activity, which was also absent in our patient [4].

Preoperative diagnosis of cauda equina paraganglioma is difficult. The diagnosis is primarily determined by pathological examination. On MRI, numerous radiological characteristics of spinal paragangliomas are common with other lumbar tumors. Therefore, paraganglioma is difficult to diagnose [1], and a wide range of differential diagnoses should be considered. This spectrum includes ependymomas specifically myxopapillary ependymoma, meningioma, schwannoma, and other relatively rare tumors such as lipoma, teratoma, and hemangioblastoma [1, 6].

Although we did not observe this condition, sympathetic paragangliomas have the potential to produce a hypertensive effect and secrete high levels of catecholamines, which can be difficult to manage intraoperatively [7]. Patients exhibiting symptoms associated with catecholamine releases, such as hypertension, tachycardia, headache, sweating, and palpitations, should undergo screening for tumor activity [7]. Vanillylmandelic acid, metanephrine, and normetanephrine can be determined in the urine [7]. This screening allows for preoperative planning for hypertensive crises.

Lumbar paraganglioma/Cauda equina neuroendocrine tumor is a slow-growing, WHO grade I tumor, and the primary treatment is surgical resection. The goal of surgery must be total resection. Fortunately, lumbar paragangliomas are generally well-encapsulated masses, facilitating gross total resection [1]. Dissection away from adherent structures is almost always possible, as in our case. After dissection, it is essential to remove the paraganglioma in its entirety [1]. Due to the highly vascular nature of these tumors, piecemeal resection poses a risk of significant bleeding and enbloc removal of the tumor is beneficial [1, 8].

Their prognosis is good if the gross total removal of the spinal paragangliomas is achieved [1]. In a recent review, there were only 5 cases of tumor recurrence in 241 cases of gross total removal (2.5%) [4]. In contrast, tumor regrowth occurred in 11 (35.5%) of 31 cases of subtotal resection [4]. Studies have failed to demonstrate any benefit of adjuvant radiotherapy following surgery in cases with subtotal resection [9, 10].

Paragangliomas should be considered in the differential diagnosis of lumbar intradural masses. If the patient has findings suggestive of catecholamine discharge, vanillylmandelic acid, metanephrine, and normetanephrine should be searched in the urine for planning for hypertensive crises during the operation. Total resection should be targeted in surgery. Adjuvant radiotherapy following surgery has not shown any benefit in subtotal resection cases.
